# A Novel Homozygous *CYP19A1* Gene Mutation: Aromatase Deficiency Mimicking Congenital Adrenal Hyperplasia in an Infant without Obvious Maternal Virilisation

**DOI:** 10.4274/jcrpe.galenos.2018.2018.0140

**Published:** 2019-05-28

**Authors:** Fatma Dursun, Serdar Ceylaner

**Affiliations:** 1 Ümraniye Training and Research Hospital, Clinic of Pediatric Endocrinology, İstanbul, Turkey; 2 Intergen Genetic Centre, Medical Geneticist, Ankara, Turkey

**Keywords:** 46, XX disorders of sex development, CYP19A1 gene, aromatase deficiency

## Abstract

Aromatase deficiency is a rare, autosomal recessive disorder in which affected patients fail to synthesize normal estrogen. Herein, we report a 46, XX patient born with virilised external genitalia. A novel homozygous mutation in the *CYP19A1* gene, causing aromatase deficiency, was detected. A 30-day infant registered as a male was referred to pediatric endocrinology because of a uterus detected on ultrasonography. The infant was born at 23 gestational weeks by C-section because of preeclampsia and premature membrane rupture. The parents were consanginenous. There was no evidence of virilisation, such as acne, hirsutism, deep voice or clitoral enlargement in the maternal history. Physical examination of the infant revealed complete scrotal fusion and a single urogenital meatus, consistent with Prader stage-3. A standard dose adrenocorticotropic hormone (ACTH) test revealed an inadequate cortisol response and high 17-hydroxy progesterone levels, suggesting simple virilising congenital adrenal hyperplasia (CAH) due to 21-hydroxylase deficiency. However, no mutation in the *CYP21A2* gene was detected. At age 2.5 years the ACTH test was repeated, after suspension of hydrocortisone treatment for 48 hours, when resulting cortisol and androgen levels were normal. The patient was re-evaluated in terms of 46, XX disorders of sex development (DSD), especially with a suspicion of aromatase deficiency. A novel, homozygous, exon 6 deletion was identified in the *CYP19A1* gene. Aromatase deficiency may be confused with CAH in the newborn period. In this case 46, XX DSD aromatase deficiency was present in the absence of a history of maternal virilisation or large and multicystic ovaries.

**What is already known on this topic?**

In aromatase deficiency, the accumulated androgens may cause signs of maternal virilisation during pregnancy. Large multiple cysts have been described in aromatase deficient girls during infancy and childhood. In previous reports of aromatase deficiency, cases were term neonates of average weight for gestational age.

**What this study adds?**

In this report, we describe a case of aromatase deficiency showing disorder of external genital development in a preterm infant born at age 23 weeks. A report a novel large deletion in the *CYP19A1* gene was shown. Maternal virilisation was not a marked finding in our case, except for a mild deep voice. The absence of virilisation in our patient’s mother could likely be due to the premature delivery of the patient.

## Introduction

Aromatase (cP450arom) catalyses the conversion of androgens to estrogens. The biological importance of aromatase is related not only to its role in estrogen biosynthesis, but also to its potential influence on the balance of the androgen-estrogen ratio in different tissues. In humans, cP450arom is encoded by a single gene *(CYP19A1)*, that is located on chromosome 15q21.1. The protein-coding sequence is contained within nine exons (E2-E10), spanning approximately 35 kb (1,2,3). The CP450arom enzyme is mainly located in the endoplasmic reticulum of estrogen-producing cells in the ovary, placenta, testis, brain, adipose tissue, liver, muscle and hair follicles (4,5).

Aromatase deficiency is a rare, autosomal recessive disorder in which affected patients do not have normal estrogen synthesis (1). During pregnancy, dehydroepiandrosterone sulphate (DHEAS) and 16OH-DHEAS, arising from the fetal adrenal gland and liver, respectively, become important sources for the synthesis of placental estrogens (4,5,6). Fetuses lacking aromatase activity are not able to convert DHEAS to estrogens in the placenta. DHEAS is therefore converted to testosterone, resulting in the virilization of both fetus and mother. Since the first description of aromatase deficiency by Shozu et al (7) in 1991, around 40 cases have been reported (1,3,4,5,7,8,9,10,11,12,13,14,15,16,17,18,19,20).

In aromatase deficiency, the accumulated androgens may cause signs of maternal virilisation (acne, deep voice, clitoral enlargement) during pregnancy. After delivery these symptoms usually disappear gradually. In the postpartum period, some clinical and laboratory findings of androgen excess regress and androgen levels return to normal levels. In most female infants exposed *in utero* to excessive androgen levels, ambiguous genitalia have been reported. Delayed skeletal maturation has been described and most affected girls have multiple ovarian cysts and failure of breast development at puberty (3,5).

Herein, we report a 46, XX patient born with virilised external genitalia. A novel homozygous mutation in the *CYP19A1* gene, causing aromatase deficiency, was detected.

## Case Report

A 30-days old infant with a male-dominant genital appearance was referred to pediatric endocrinology because of a uterus, detected on ultrasonography. The infant was born at 23 weeks of gestation by C-section because of preeclampsia and premature membrane rupture. The parents were consanginenous. Birth weight was 680 gr. The infant was intubated, given surfactant treatment and required mechanical ventilation support. Bilateral cryptorchidism and hypospadias were thought to be associated with the severe prematurity. Since gender assessment at birth was made as male, the baby received a male name and identity card. He was the first baby of a 25-year old healthy mother and a 27-year old healthy father who were first cousins. The mother had had two abortions in the past, so she was treated with progesterone for one month between the 16^th^ and 20^th^ gestational weeks and also with salicylic acid throughout the pregnancy. There was no evidence of virilisation, such as acne, hirsutism, deep voice or clitoral enlargement in the maternal history. Physical examination of the infant revealed complete labioscrotal fusion and a single urogenital meatus, consistent with Prader stage-3. Gonads were not palpable, a chorda was present and the phallus was measured as 2x1 cm on the dorsal and 1.6x1 cm on the ventral side. At the time of the investigation the patient was still being followed in the neonatal intensive care unit and having mechanical respiratory support. On postnatal day 30, the patient’s hormone levels were as follows: 17-hydroxy progesterone (17OHP): 41 ng/mL [normal limits (NL) <35.5 ng/mL], DHEA sulphate (DHEASO4): 1500 µg/dL (NL 123-882 µg/dL), testosterone: 2.94 ng/mL (NL 0.05-0.16 ng/mL), FSH: 1.3 IU/L (NL 0.3-2.6 IU/L), LH: 0.48 IU/L (NL 0.1-8.5 IU/L), estradiol <10 pg/mL (NL <15 pg/mL), progesterone: 4.7 ng/mL (NL 0.18-6.4 ng/mL). Karyotype was 46, XX. A standard dose adrenocorticotropic hormone (ACTH) test (30 µg/kg/dose) revealed an inadequate stimulated cortisol and high 17OHP levels, suggesting simple virilising congenital adrenal hyperplasia (CAH) likely due to 21-hydroxylase deficiency (Table 1). Additionally there were several other problems, such as septicemia, surfactant deficiency and respiratory distress. The patient was on mechanical ventilation due to severe prematurity at this time. Although the classical findings of adrenal insufficiency were not present, the decision was taken to administer hydrocortisone® replacement since cortisol deficiency could not be excluded. Hydrocortisone® was commenced at a dose of 10 mg/m2/day, three times a day. The name and identity card of the baby were changed to female with the agreement of the parents and the decision of multidisciplinary gender assessment committee.

**Figure 1:**
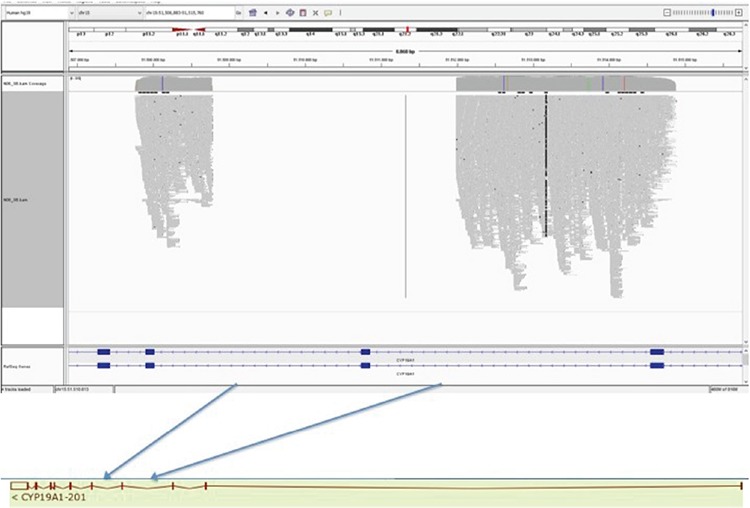
Identification of deletion in next generation sequencing, as visualized by integrative genomics viewer software. A 3212 bp deletion represented by blue arrows, was detected within chr15: 51.511.985–51.508.774 (NM_000103.3:c.629-1453_744-486del)

Over the next two years, androgen levels were quite low, despite hydrocortisone doses as low as 6-7 mg/m2/day, and no mutation in *CYP21A2* gene was detected. This unusual clinical condition and lack of a mutation in *CYP21A2* gene led to doubt concerning the security of the diagnosis of 21-hydroxylase deficiency. At the age of two years and six months the standard dose ACTH test was repeated, after suspension of hydrocortisone treatment for 48 hours. The results of this test showed the cortisol and androgen levels were normal (Table 1). When maternal history was re-evaluated, the mother remembered that she had a mild deep voice during pregnancy. The patient was re-evaluated in terms of 46, XX disorders of sex development (DSD), especially with the suspicion of aromatase deficiency (Table 2). Finally, aromatase deficiency was confirmed by genetic analysis (Figure 1).

At the last clinical visit, the patient was 4.3 years old, height was 95.5 cm (-2.3 SD), weight 14.5 kg (-1.27 SD) and breast development was Tanner stage-1. Further examinations were performed for disorders which could be associated with aromatase deficiency (Table 2).

Informed consent was obtained from the parents of the patient for publication of this case.

### Genetic Analysis

An Ethylenediaminetetraacetic acid blood sample was taken for *CYP19A1* gene sequence analysis. At the PCR step, as the very large region including exon 6 could not be amplified, a long PCR and sequence analysis was planned to detect exact breakpoints. Sequence analysis with a Next Generation Sequencing Method (Illumina-MISEQ, San Diego, CA, USA) was done and a 3212 bp deletion within chromosome 15:51.511.985-51.508.774 was detected (NM_000103.3:c.629-1453_744-486del). This large deletion was evaluated as a likely “pathogenic” variant due to ACMG criteria.

The *CYP19A1* gene contains 10 exons and exon 6 was largely deleted with some parts of introns of both sites and two canonical splice sites. This was a null variant. The allele was not found in gnomAD exomes. This is a conserved region in different species. This was a novel variant.

## Discussion

In this report, we describe a case of aromatase deficiency in a 23-week preterm infant with a disorder of external genital development. We report a novel large deletion in the *CYP19A1* gene (Figure 1). To date, more than 33 different mutations in the *CYP19A1* gene have been reported in patients with aromatase deficiency. These mutations include mis-sense, splice site, non-sense, insertions and small deletions and one large intragenic deletion (1,3,4,5,8,9,10,11,12,13,14,15,16,17,18,19,20,21,22). The majority of the mutations reported are located in exons 9 and 10, which encode the substrate-binding site and haem-binding domains, respectively (12). Our patient had a large deletion in exon 6. Although we did not make a functional study, this variant is a null variant and classified as a likely pathogenic variant due to ACMG criteria. The fact that we were not able to conduct a functional study related to the mutation we identified was the limiting factor of our study.

Aromatase deficiency causes virilisation (acne, deep voice, clitorial enlargement) in the mother because placental androgens cannot be converted to estrogens, resulting in excessive androgen levels and virilisation of the mother during pregnancy (16). While it is an important clue, maternal virilisation is not a rule. In the study of Marino et al (3), three of six cases had a history of gestational virilisation. Maternal virilisation was not a marked finding in our case, except for a mild deep voice. Why some mothers do not have signs of virilisation can be explained by either a lower fetal adrenal androgen secretion or adequate placental estrogen production (16). Grumbach and Auchus (23) reported that a placental aromatase activity as low as 1% of normal is enough to prevent maternal virilisation. Furthermore, substantial placental estrogens are produced, especially in the 3^rd^ trimester of gestation (16). The absence of virilisation in our patient’s mother could likely be due to premature delivery or to the presence of partial aromatase activity.

In most female cases of aromatase deficiency, disorders of external genitalia with various degrees of masculinization have been reported. Gonads were non-palpable and internal genitalia differentiation was normal female in these cases (1,3,4,5). For this reason, these patients can be diagnosed as CAH, which is the most common cause of virilisation in a female fetus (1). Similarly, our patient was considered to have CAH because of physical findings and also because of quite high androgen levels. In addition the severe prematurity and the lack of data about normal androgen levels in such neonates led to initial diagnostic confusion in our patient. Low androgen levels, despite low hydrocortisone doses on the follow-up, are very unusual in classical CAH patients. This important observation, together with the lack of mutation in *CYP21A2*, encouraged us to reconsider a diagnosis of 21-hydroxylase deficiency.

A clinical phenotype, including changes in the hypothalamic-pituitary-gonadal axis, ovarian cyst development, skeletal maturation and growth, as well as changes in insulin sensitivity and lipid profile, has been reported in aromatase deficiency (5). Marino et al (3) investigated the hypothalamic-pituitary-gonadal axis and described high levels of luteinizing hormone (LH) and follicle-stimulating hormone (FSH) in the neonatal period. A two-month old girl, reported by Mullis et al (17), had elevated FSH levels (baseline and GnRH-stimulated) but normal LH levels (baseline and GnRH-stimulated). Contrary to these findings, our patient had normal gonadotropin levels on postnatal day 60 (Table 2). LH and FSH levels were elevated at the ages of 26 and 52 months (Table 2). In previous reports of aromatase deficiency, cases were term neonates with average weights for gestational age (5,7,17,18). Our patient was born at the 23rd week of gestation, thus gonadotropin levels in early infancy would probably not be helpful in the diagnosis. In premature infants without aromatase deficiency, gonadotrophin levels are very high after birth, but a sharp decrease in FSH levels is seen around term age. Also, in term neonate without aromatase deficiency, gonadotrophin levels are low at birth and increase progressively afterwards (24,25). Since we measured gonadotropin levels at a near term-equivalent age, it might have been coincidental with the time of rapid decrease.

Large multiple cysts have been described in aromatase deficient girls during infancy and childhood due to chronic stimulation by gonadotropins that cannot be suppressed because of estrogen deficiency (3,5,17,18). Marino et al (3) reported a case series of five patients. Four of them, aged 18, 7, 12 and 10 years, had increased ovarian size with large cysts. They were at a pubertal stage except for the 7-year old. Only one, a 3-year old girl, had normal ovaries. There are some patients who do not have large cysts and may even have hypoplastic ovaries. To date, seven patients with hypoplastic ovaries have been reported (4,12,16,19,20). Thus, there is no consistent ovarian phenotype in patients with aromatase deficiency since some were reported to have large and polycystic ovaries, while others had normal ovarian morphology (16). Despite quite elevated levels of FSH, we did not observe any ovarian cysts in periodic ultrasonographic screening of our patient and she continues to show normal ovarian morphology.

Little is known about the bone phenotype of girls with aromatase deficiency (26). It is accepted that estrogens are important in preserving adequate bone mineral density (BMD) (5). However, data on the role of estrogens on bone mineralization during childhood are scarce. Janner et al (26) found decreased BMD in a 3.5-year old patient, while Belgorosky et al (18) reported normal BMD in a 6-year old patient. It may be true that some expression of cP450arom protein might be enough to maintain a normal mineral bone density. On the other hand, men with aromatase deficiency show a distinct bone phenotype characterized by osteopenia (27). We performed a BMD measurement in our patient at the age of four years, revealing osteopenia (-1.4 SDS) when re-calculated for the patient’s height age.

The usefulness of estrogen treatment during infancy and childhood in affected female patients is not clear. Mullis et al (17) reported that low doses of estradiol in a 3-year-old affected girl resulted in normalization of serum gonadotropins, regression of enlarged ovaries and improvement in BMD. Janner et al (26) showed the positive impact of oral 17-β estradiol treatment on longitudinal growth, bone age maturation, regulation of pituitary gonadotropin feedback, improving multicystic ovaries and bone density in the long-term follow-up of a girl with a compound heterozygote mutation in *CYP19A1* gene. So far, our patient has not been started on estrogen treatment, since her ovaries are still normal and existing data on estrogen treatment for these patients are inadequate.

In conclusion, a novel mutation in *CYP19A1* gene explains the virilisation of our patient. Aromatase deficiency could easily be confused with CAH, especially in a preterm infant. In a case of 46, XX DSD, aromatase deficiency can present without a history of maternal virilisation or in the absence of large and multycystic ovaries. The absence of virilisation in our patient’s mother could likely be due to premature delivery of the patient or presence of partial aromatase activity. It is not clear if premature delivery and aromatase deficiency are related or are coincidental in our patient. The effect of inadequate androgen-estrogen conversion in placenta on the continuation of the gestational process in aromatase deficiency is not clear. In our opinion further investigation of this relationship and possible mechanisms are warranted.

This case report emphasizes the importance of considering aromatase deficiency as a very rare cause of 46, XX DSD and the need to perform genetic analyses in patients, especially in the absence of a definite diagnosis. Further published cases will enhance our knowledge of the phenotypic spectrum of aromatase deficiency.

## Figures and Tables

**Table 1:**
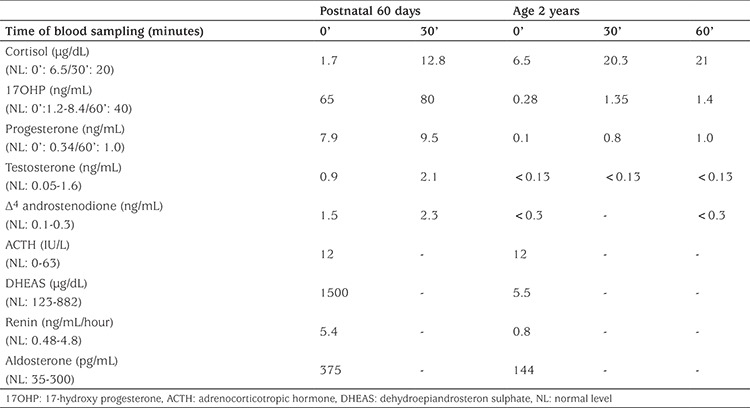
Results of classical adrenocorticotropic hormone stimulation test at ages 60 days and 2 years

**Table 2:**
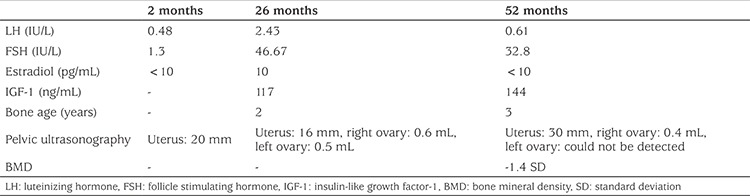
Laboratory findings of the patient at diagnosis and follow-up
